# Supplementary material for: Acquired CRISPR spacers and rhamnose-glucose polysaccharide defects confer resistance to Streptococcus mutans phage ɸAPCM01
Source: Microbiology (Reading). 2025 Jun 19;171(6):001575. doi: 10.1099/mic.0.001575 (PMC12178566; doi:10.1099/mic.0.001575)
Supplement: Uncited Supplementary Material 1. [file mic-171-01575-s001.pdf]

Supplemental Material

Title: Acquired CRISPR spacers and rhamnose-glucose polysaccharide defects confer resistance to *Streptococcus mutans* phage ϕAPCM01

Lucas A. Wall and Daniel Wall

Department of Molecular Biology, University of Wyoming, Laramie, WY 82071, USA

Table S1 Endogenous CRISPR spacers in host DPC6143

Spacer	Spacer sequence	Phage/species hit	Identity
1	AAAATGCTAGTTGAAACA<br>TTTTCTAGTTTT	Phage M102	28/30 bp
2	CGATATTGGAACAACTGT<br>TACAACTCCAAC	*Bacillus cytoxillus* strain E28	28/30 bp
3	TGGAACTGTCCAGAATTG<br>CAATAGCAGCAC	No relevant hit	
4	TGTCTATCTAAAAAAAGA<br>TAATGGTGAAAA	No relevant hit	
5	GTAAAAAACGGAGAGTTG<br>TATGCATATTTT	No relevant hit	
6	TCCGAGAATAGGAGCTT<br>GACAGTTCCCGTC	No relevant hit	
7	AAATTTCTTATTAAGAAAA<br>GTGAGATGAAA	No relevant hit	
8	CAGCAAGAACGCAGTAG<br>TTTATGTAGGGGG	Phage ϕAPCM01	27/30 bp
9	TAGACGCAGAACTATTAG<br>TTTTGAGGATAC	No relevant hit	
10	CGCTAGTAATTAATGGTA<br>AACAGCATAATA	*Streptococcus* phage phiKSM96	23/30 bp
11	TAATGAGAAAGTCATAGT<br>CCATGTCTACCG	No relevant hit	
12	GAGAAAGCAGAAAATACT<br>TCTATTGGTTCT	*Streptococcus* phage phiKSM96<br>*S. mutans* KCOM 1054 (spacer)	29/30 bp<br>30/30 bp
13	GCCGTCAGAGTTCTTCCA<br>CTCTTGCTCT	Phage *Caudoviricetes* sp. ctNo011	30/30 bp
14	CTCCAATTGAAAATATTG<br>ATCCAGGTTATT	Phage *Caudoviricetes* sp. ctNo011<br>*S. mutans* KCOM 1054 (spacer)	28/30 bp<br>30/30 bp
15	AGCGAAATTGGAAGATGT<br>TTAGTGTTGCTT	No relevant hit	
16	ATAGTACGAAAAATGAAA<br>ATTTAGGAGTTC	*S. mutans* UA159 and *S. mutans* KCOM<br>1054 (spacers)	30/30 bp
17	TTGCTAAAAAAGGTGATG<br>TGGTTTATAAGT	*Candidatus Pelagibacter giovannonii* NP1	27/30 bp
18	AGCTATTAAACGTGGTTT<br>AACACAATCTTG	No relevant hits	
19	CTATTGCTGTCTTGTAGT<br>GCTTCGAGTCAA	Phage *Caudoviricetes* sp. ctQS92	24/30 bp
20	TACTAAGCAGTTTGCAGA<br>TGAAGACCCGCG	Phages M102 & M102AD	27/30 bp
21	CCTTTTGACCTGACTAAC<br>CAGGAGCAAGAG	*S. mutans* phage smHBZ8	27/30 bp
22	TAATTCGTCAAATTCTCC<br>ACCCAGAAGCG	Phage M102	28/29 bp
23	ACCGCGTTGTTCTAAAAA<br>TCTAATTTGTTT	*S. mutans* KCOM 1054 (spacer)	29/30 bp
24	TGGACGCAAGAATATCTA<br>TTTAAATGGTTC	No relevant hits	
25	AAATCCACCAACTGACTC<br>AATTGATCCATT	No relevant hits	
26	CCAAAATTTTTAGCTCAA<br>AAATACGGCAAA	Phages ϕAPCM01 & smHBZ8<br>Phage M102AD	28/30 bp<br>23/30 bp
27	AAAGCTGATTTTTTTATTT<br>TTCCAGCCAGT	*Streptococcus dysgalactiae* subsp. *equisimilis*<br>strain MGGS36030	30/30 bp
28	AATAGAATCAAATTCTCC<br>TAAGTCAGTTAA	*S. mutans* and *Streptococcus* spp.	30/30 bp

Mismatched bases with ϕAPCM01 underlined.

Table S2 Mutations in ϕAPCM01 resistant isolates

Strains	Protein/element	Mutations	RefSeq	DNA coordinates
DPC6143 (WT)	N/A	N/A	N/A	
rgpX$^{W190NS-CR1}$	RgpX<br>CRISPR spacer<br>ZupT<br>*serP2-I-rqcH*	W190NS<br>CRISPR 1<br>M48I<br>Intergenic G->T	WP_002301943.1<br>N/A<br>WP_002262376.1<br>N/A	1,246,166<br>753136-753166<br>1,986,979<br>702,516
rgpX$^{P521H-CR2ab}$	RgpX<br>CRISPR spacer<br>CRISPR spacer	P521H<br>CRISPR 2a<br>CRISPR 2b	WP_002301943.1<br>N/A<br>N/A	1,245,173<br>753136-753166<br>753992-754027
rgpF^{E491K}	RgpF<br>Glycoside hydrolase<br>Hypo peptidase	E491K<br>G249R<br>A166S	WP_002261980.1<br>WP_002263319.1<br>WP_019803208.1	1,250,491<br>1,365,248<br>1,525,118
rgpF$^{T419P-CR3ab}$	RgpF<br>CRISPR spacer<br>CRISPR spacer<br>Glycoside hydrolase	T419P<br>CRISPR 3a<br>CRISPR 3b<br>G249R	WP_002261980.1<br>N/A<br>N/A<br>WP_002263319.1	1,250,707<br>753136-753166<br>754322-754357<br>1,365,248
rgpX^{FS}	RgpX<br>Hypo zinc-binding alcohol dehydrogenase	ΔG codon 194<br>+A insertion	WP_002301943.1<br>WP_019802715.1	1,246,154<br>994,472
rgpF$^{G382S-A}$	RgpF<br>Glycoside hydrolase	G382S<br>G249R	WP_002261980.1<br>WP_002263319.1	1,250,818<br>1,365,248
rgpF$^{G382S-B}$	RgpF<br>Glycoside hydrolase<br>tRNA<br>tRNA<br>23S ribosomal RNA<br>16S ribosomal RNA	G382S<br>G249R<br>T->G<br>Deletion<br>Multiple mutations<br>Multiple mutations	WP_002261980.1<br>WP_002263319.1<br>N/A<br>N/A<br>N/AN/A	1,250,818<br>1,365,248<br>22,362<br>22,386<br>1,862,014-1,863,946<br>1,864,804-1,865,996
rgpD^{F162Y}	RgpD	F162Y	WP_002267627.1	1,254,111
CR4	CRISPR spacer	CRISPR 4	N/A	753135-753164
CR5abc	CRISPR spacer<br>CRISPR spacer<br>CRISPR spacer	CRISPR 5a<br>CRISPR 5b<br>CRISPR 5c	N/A<br>N/A<br>N/A	753172-753268<br>753701-753928<br>754520-754555
CR6ab	CRISPR spacer<br>CRISPR spacer<br>Glycoside hydrolase	CRISPR 6a<br>CRISPR 6b<br>G249R	N/A<br>N/A<br>WP_002263319.1	753136-754555<br>754585-754620<br>1,365,248
CR7	CRISPR spacer<br>Hypo peptidase-I-ccpA	CRISPR 7<br>G->T	N/A<br>N/A	753134-753171<br>546,203
CR8ab	CRISPR spacer<br>CRISPR spacer	CRISPR 8a<br>CRISPR 8b	N/A<br>N/A	753136-753766<br>754520-754620
CR9abc	CRISPR spacer<br>CRISPR spacer<br>CRISPR spacer<br>Hypo<br>Glycoside hydrolase	CRISPR 9a<br>CRISPR 9b<br>CRISPR 9c<br>L122R<br>G249R	N/A<br>N/A<br>N/A<br>WP_002273056.1<br>WP_002263319.1	753136-753171<br>753136-753171<br>753991-754028<br>582,063<br>1,365,248
CR10	CRISPR spacer	CRISPR 10	N/A	753135-754159

Hypo, hypothetical; N/A, not applicable.

Table S3 DNA sequence of acquired spacers and ϕAPCM01 targets

Strain	CRISPR ID	bp	PAM (5'-3')	Spacer sequences (5'-3')	Phage gene product
rgpX$^{W190NS\text{-}CR1}$	CRISPR 1	30	ACCA	TTGCTAAGAACGACGGCACAGCAAGCGCTA	Minor tail protein
rgpX$^{P521H\text{-}CR2ab}$	CRISPR 2a<br>CRISPR 2b	30<br>30	ACCC<br>GCCC	GAAACACAGAGCTAGTCCGCAAGAGCTACA<br>GTTTACGCTGTAATCATTTTGATTGTAATT	RecT-like-ssDNA protein<br>Portal Protein
rgpF$^{T419P\text{-}CR3ab}$	CRISPR 3a<br>CRISPR 3b	30<br>30	TCCA<br>ACCT	AACTATGCAGTAACTCCCGACCCCGTGTGG<br>GCAATAATGGAAGTTGCTGGTGAAATGACC	Endolysin<br>Tape measure protein
CR4	CRISPR 4	29	CCGC	CGCCGTTGATATTAATAGTCATTGTTGCG	Phage structural protein
CR5abc	CRISPR 5a<br>CRISPR 5b<br>CRISPR 5c	30<br>30<br>30	TCCA<br>ACCT<br>GCCG	TTATTGCAGGTTTCATTTTAGGAAACGTG<br>GTGTGTCCGAAAGGATGACCGTAAACGGTA<br>GCCGTATACAGTATCAAGTGCATAGACAGT	Tape measure protein<br>Endolysin<br>Terminase large subunit
CR6ab	CRISPR 6a<br>CRISPR 6b	30<br>30	GCCA<br>CCCT	TGTTGAGCTGAGCCAGGAGTTACTCCGCCG<br>GAAACACACAAAGCAAAAACGAAAGCAAAC	Major tail protein<br>Major tail protein
CR7	CRISPR7	30	ACCT	CTTGTAAGTCTCTGATGTCAATCTCATCTA	Hypothetical protein
CR8ab	CRISPR 8a<br>CRISPR 8b	30<br>30	ACCA<br>ACCC	GCGGGCTCAACTTGATCCGTGGCGCACTTG<br>ATCAGCTCCACAAATCAGCTTTAATTTTAA	Tape measure protein<br>Major tail protein
CR9abc	CRISPR 9a<br>CRISPR 9b<br>CRISPR 9c	30<br>30<br>29	ACCA<br>CCCA<br>CCAA	GACTATATCTTTTTCTTTGTGTTTAATTAA<br>TCTGGTTTTGAACCAATAAATTTATAAGGC<br>TGGTTTAGCCATGATTTCTCCTCTTTTCG	Intergenic<br>Distal tail protein<br>Hypothetical protein
CR10	CRISPR 10	29	CCGC	CAATCTCTAAGCGTTGGTGCTCAATGGAA	Capsid protein

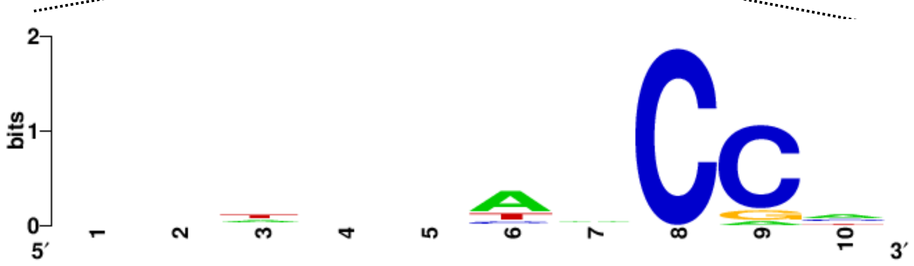

Conserved CC PAM sequences underlined. WebLogo of the ten nucleotides upstream of the 5' end of protospacers.

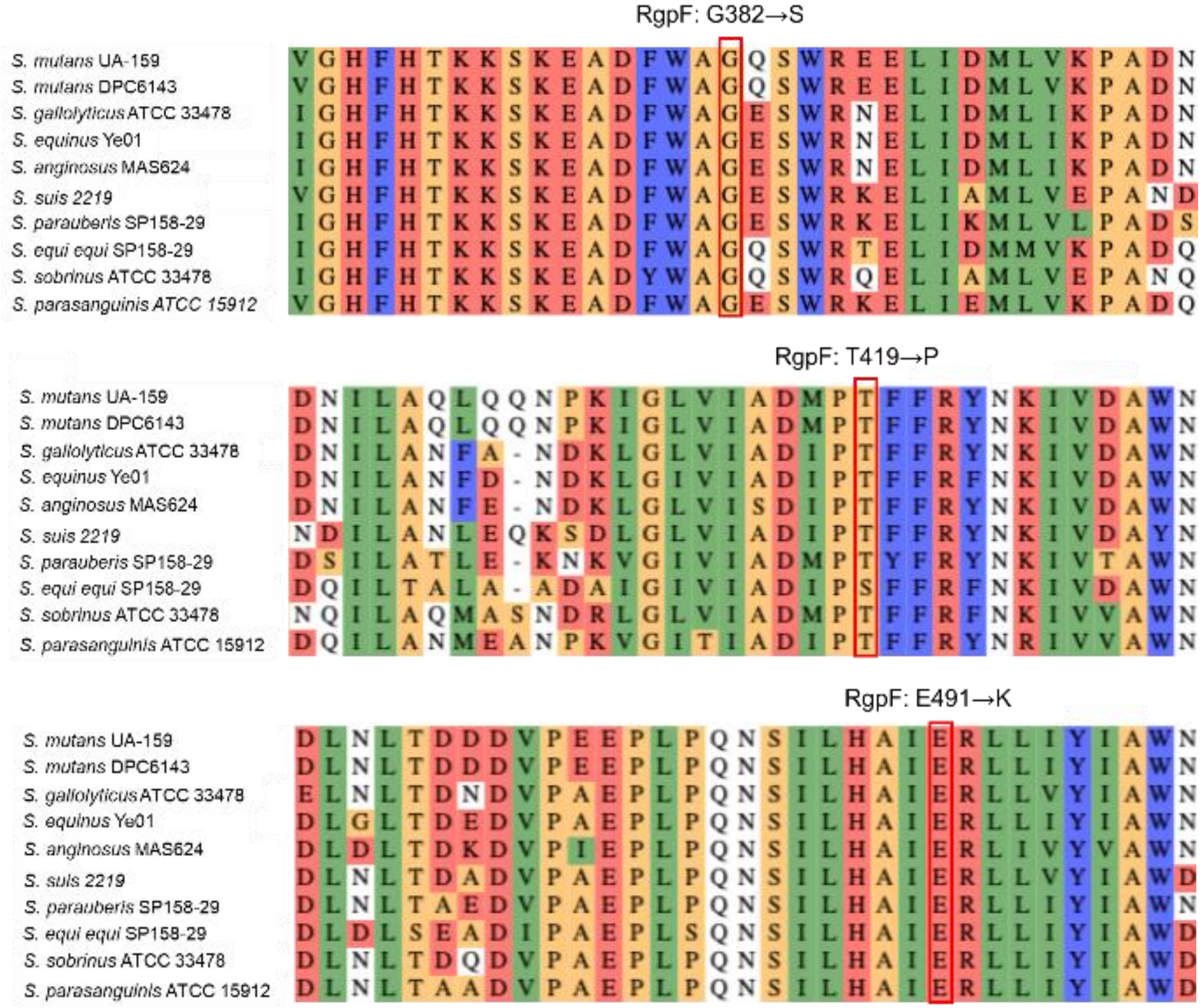

Figure S1. Sequence conservation among *Streptococcus* species around *S. mutans* RgpF substituted residues that confer ϕAPCM01 resistance.

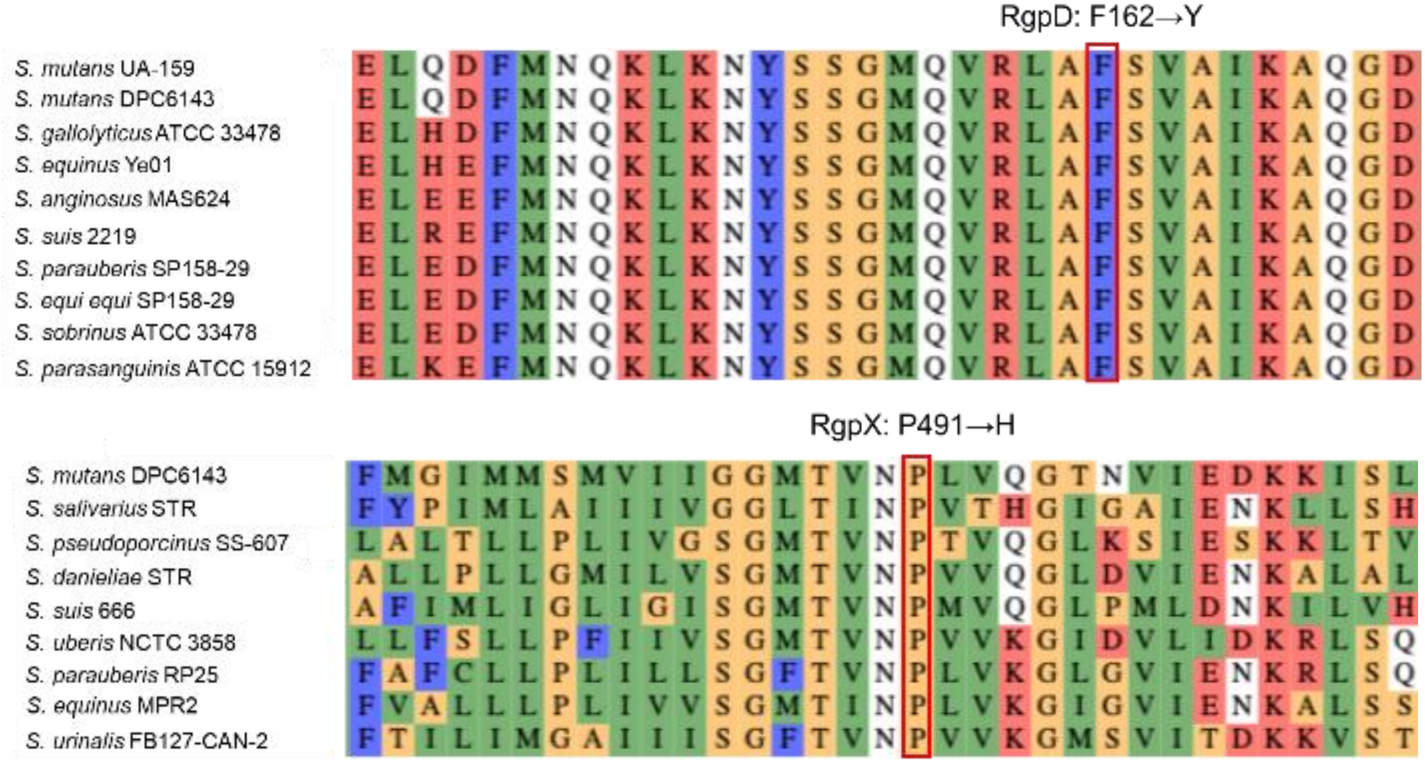

Figure S2. Sequence conservation among *Streptococcus* species around *S. mutans* RgpD and RgpX substituted residues that confer ϕAPCM01 resistance.

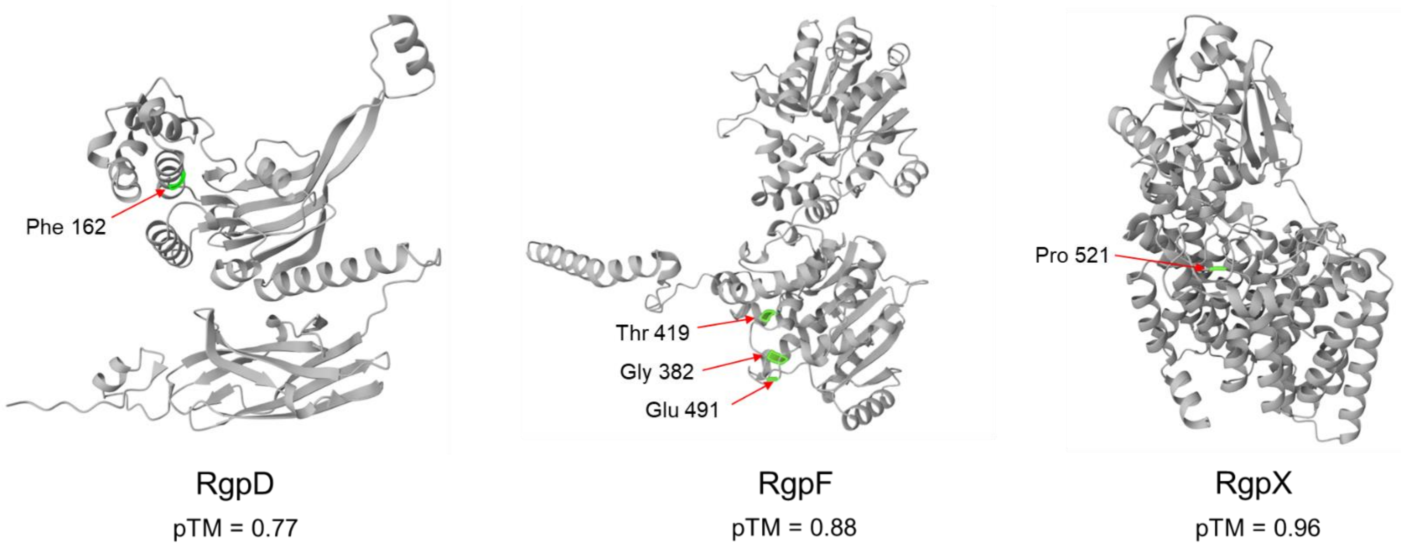

Figure S3. AlphaFold 3 predicted structures of Rgp proteins. Residues with substitutions that confer ϕAPCM01 resistance highlighted. All pTM scores indicate high confidence structures.

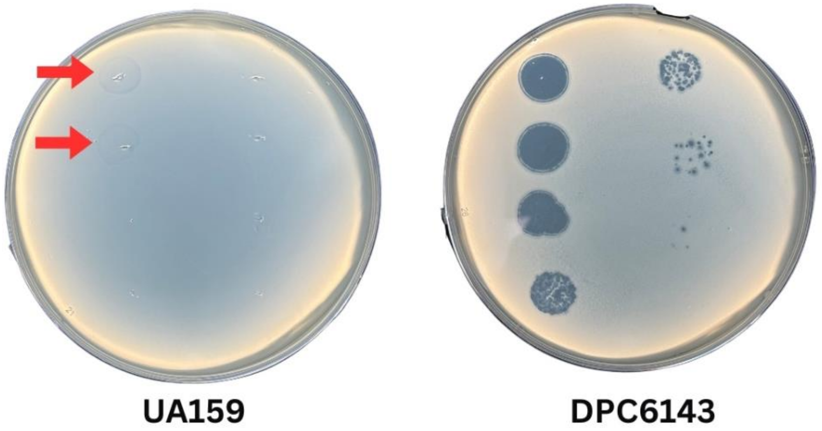

Figure S4. Serial dilutions of APCM01 on UA159 or DPC6143 lawns. Faint clearings observed on UA159 (arrows), whereas individual plaques seen on DPC6143.

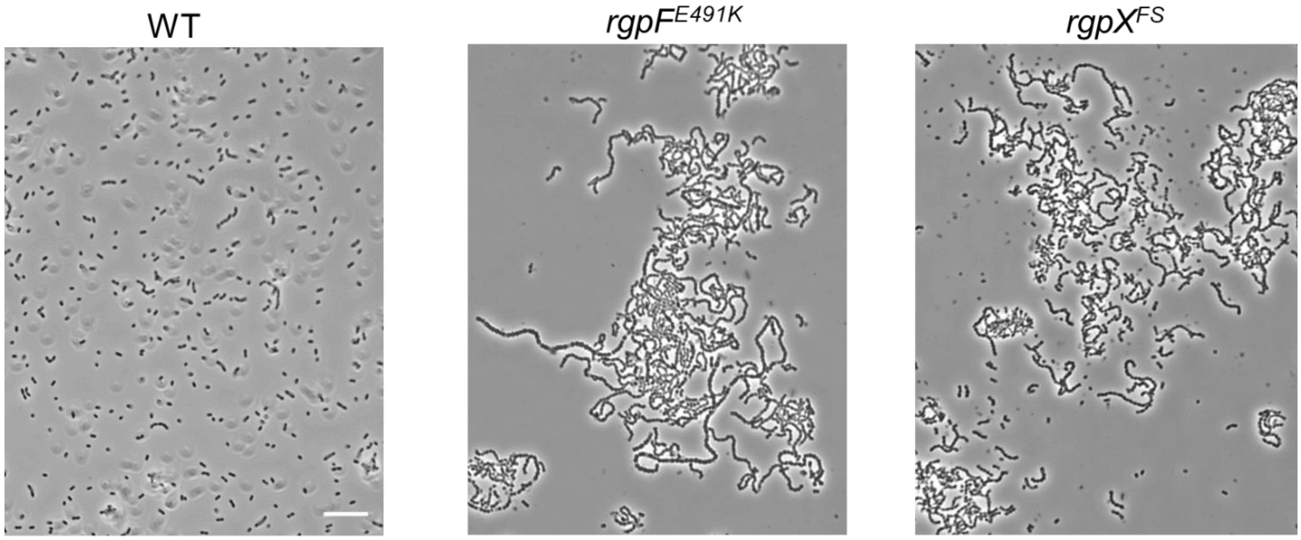

Figure S5. Culture tube sediment from Figure 6A. Scale bar, 10 µM.
